# A model of indoor thermal condition based on traditional acehnese houses using artificial neural network

**DOI:** 10.1016/j.heliyon.2024.e40644

**Published:** 2024-11-26

**Authors:** Muslimsyah, Abdul Munir, Yuwaldi Away, Abdullah, Teuku Yuliar Arif, Andri Novandri

**Affiliations:** a Doctoral Program, School of Engineering, Universitas Syiah Kuala, Banda Aceh, 23111, Indonesia; b Department of Architecture and Planning, Universitas Syiah Kuala, Banda Aceh, 23111, Indonesia; c Department of Electrical and Computer Engineering, Universitas Syiah Kuala, Banda Aceh, 23111, Indonesia; d Department of Civil Engineering, Universitas Syiah Kuala, Banda Aceh, 23111, Indonesia; e Department of Computer Engineering, Universitas Serambi Mekkah, Banda Aceh, 23249, Indonesia

**Keywords:** Thermal condition, Rumoh aceh, ANN, Orientation, Region

## Abstract

This paper examines the thermal condition aspects of traditional Acehnese houses (Rumoh Aceh) in Indonesia. This study highlights the importance of considering traditional architecture in achieving comfortable living conditions. Through the integration of cultural values, architectural design, and environmental factors, this research evaluates the prediction of thermal conditions within Rumoh Aceh based on building orientation and location, serving as guidelines for architects in designing buildings in the Aceh region. The study employs an ANN (Artificial Neural Network) algorithm to predict thermal condition parameters including indoor: temperature, humidity, and wind speed. The sample data used to train the ANN model consists of thermal data from five different rooms and meteorological data. The ANN model is employed to predict the thermal conditions in the Rumoh Aceh building based on its orientation and location. In this study, two models were developed, namely MODEL_01, for predicting indoor temperature and humidity, and MODEL_02, for predicting indoor wind speed. In MODEL_01, the error tolerance level obtained is ±1.05, and in MODEL_02, it is ±0.03. The prediction results based on the building orientation show the lowest average indoor temperature value of 29.19 °C, occurring at an angle of 292.19°. Furthermore, the lowest average indoor humidity value is 74.57 %, which also occurs at an angle of 292.19°. However, for indoor wind speed, the angle of 260° records the lowest average value of 0.125 m/s, while the highest average value is observed at 292.19°, at 0.132 m/s. The prediction results based on the building's location show the lowest average temperature value, which is 29.44 °C in the Aceh Besar region. Furthermore, the lowest average humidity value is 75.11 % in the North Aceh region. Meanwhile, the highest average wind speed value is 0.174 m/s in the Sabang region. This study reveals that at the orientation angle of 292.19° (the qibla direction), it produces optimal thermal conditions in terms of temperature and humidity with low wind speed. Other findings indicate that the Aceh Besar, North Aceh, and Sabang regions have optimal thermal conditions based on temperature, humidity, and wind speed.

## 1 Introduction

Traditional houses are a type of residential building that follows the traditional architectural style that has been a part of a society's cultural heritage for many years. Traditional houses reflect the cultural identity of a region, which is the result of the local community's adaptation to environmental and climatic challenges [1]. The design of traditional houses, including their form, materials, and layout, is selected based on knowledge of the local environmental conditions [[2], [3], [4]]. One crucial aspect that receives significant attention is thermal condition. Optimal thermal conditions create a comfortable living environment, particularly in the face of climate fluctuations and diverse environmental situations. One key element in achieving optimal thermal conditions is air circulation [[5], [6], [7]]. Window openings, gaps and ventilation holes in walls and roofs, and wind-catcher designs are a few strategic examples used to establish proper air circulation. Appropriate ventilation allows airflow into the interior spaces, maintaining a cool temperature, and reducing humidity levels [[8], [9], [10]]. The use of suitable building materials also contributes to enhancing thermal quality. Building materials like stone, clay, bamboo, and wood are often utilized due to their favorable thermal properties in regulating room temperatures [[11], [12], [13]]. These materials can absorb heat during the day and release it at night, creating a more stable climate within the house [14]. Building orientation also becomes a significant consideration in traditional house architectural techniques. The position and direction of the building must be considered to maximize sunlight and wind utilization [15]. Consequently, the need for room cooling during the day can be minimized, and sunlight can be harnessed as a source of natural light.

Methods to measure the level of thermal condition quality generally employ empirical models based on human assessments [16,17]. However, these methods often fail to depict the complex interaction between thermal conditions and environmental factors. Recent research indicates that empirical models, such as the PMV (Predicted Mean Vote) model and the Adaptive Comfort model, have limitations under various climatic conditions [18,19]. For instance, the PMV model often overlooks the variability of environmental factors like natural wind speed and direct solar radiation [20]. Additionally, this model is less responsive to dynamic changes in thermal conditions [21]. Studies presented in papers [22,23] suggest that a more holistic and dynamic approach is needed to represent thermal conditions more accurately. With technological advancements, the ANN (Artificial Neural Network) method has been employed as an effective tool to predict thermal condition data [[24], [25], [26]]. This process utilizes various environmental variables such as air temperature, humidity, wind speed, rainfall, and solar radiation intensity [27,28]. By utilizing data collected from various indoor and outdoor measurements, ANN can learn to recognize patterns associated with different levels of thermal condition quality. As a result, ANN can identify conditions that provide comfortable living experiences and can be used to predict the level of thermal quality based on the current environmental conditions [[29], [30], [31], [32], [33]].

Research on thermal conditions in traditional buildings from various tropical regions has been conducted, as seen in the paper [34], which discusses the thermal conditions of traditional houses in the city of Yazd, Iran, characterized by a hot dry climate. This study revealed that the thermal conditions inside the houses were considered uncomfortable due to the hot temperature indoors. As a result, traditional buildings in that area have now been equipped with cooling systems and mechanical ventilation to enhance thermal quality. Furthermore, in the paper [35], the performance of natural air ventilation design in traditional Malay houses in Penang, Malaysia, is discussed. The analysis results indicate that traditional houses are designed with an integrated system of natural air ventilation. The temperature and humidity inside these houses are slightly lower than in the outdoor area, but the indoor wind speed tends to be lower than outside. Additionally, in the paper [36] an attempt was made to evaluate the thermal conditions of traditional houses in a hot-humid climate area in the city of Bushehr, Iran. The study findings revealed that using light colors on the exterior parts of the building can help reduce hot air temperatures inside the rooms. Moreover, the use of materials with low thermal conductivity, such as wood for the ceiling and windows of the building, can contribute to improving thermal conditions. Wood has good insulation properties, thereby helping to maintain room temperatures in a stable manner.

The country of Indonesia possesses various types of traditional houses that are spread across different regions, one of which can be found in the Aceh region known as Rumoh Aceh. These structures reflect the local wisdom of the Acehnese people in coping with the hot and humid tropical climate of the area [37,38]. The distinctive feature of Rumoh Aceh lies in its unique form, elevated on stilts with rounded wooden supports. The architectural technique of Rumoh Aceh adapts to the surrounding natural environment, featuring a design with 16 vertically arranged wooden columns that provide a sense of beauty and harmony [39]. Furthermore, this structure serves as protection against the frequent floods in the region. The building also features a pyramid-shaped roof with towering shields. These roof shields function to shield the building from the sun's heat and rain while facilitating proper airflow within the house. Additionally, the walls of the building, made from wood, are a characteristic of Rumoh Aceh, helping to maintain a stable temperature within the house. This architecture has undergone centuries of adaptation, enabling it to withstand the challenges of the hot and humid tropical climate [[40], [41], [42]]. Several studies have examined the thermal conditions of Rumoh Aceh, as discussed in the paper [43]. The results of these studies revealed that the use of modern materials like corrugated zinc roofing can increase heat at certain times. Furthermore, openings designed with cross-ventilation concepts and building orientations that face the direction of the wind can be utilized as passive cooling strategies [44]. Moreover, the paper [45] presents an analysis of standard thermal conditions in Rumoh Aceh based on a comparison of three case studies: OH (Original House), ARH (Adaptive Reuse House), and AMH (Aceh Modified House). Measurement results from the three case studies indicated that the room temperature in AMH is hotter than the other two case studies. This is due to a change in the roofing material from traditional thatch to zinc, which has resulted in reduced airflow circulation.

In this paper, a model is proposed with the ability to generate data related to the thermal conditions of Rumoh Aceh. The development process of this model involves the use of the ANN method, which is an advanced learning technique. As discussed in the paper [46] which developed a thermal control strategy to manage temperature and humidity in residential homes, the findings from this research reveal that ANN-based control logic is superior in managing thermal quality according to desired conditions compared to conventional control logic. Furthermore, in the paper [47] a comparison was made between ANN and multiple regression models to estimate indoor building room temperature and relative humidity. The results indicate that the ANN model outperforms the multiple regression model. ANN models can be useful for evaluating indoor thermal conditions and determining appropriate sizes for HVAC (Heating, Ventilation, and Air Conditioning) systems. Based on this literature, it can be concluded that ANN algorithms have the ability to learn and recognize complex patterns in data, including environmental factors that can influence thermal conditions. This allows ANN to model more complex and accurate relationships between environmental conditions and thermal quality levels. The ANN algorithm has been widely utilized in various studies related to predicting the level of thermal conditions in residential homes. Several pieces of literature have proposed different ANN models based on environmental data and housing conditions, as illustrated in Table 1. Based on the literature, it can be concluded that the thermal conditions indoors are influenced by various external and internal factors, including indoor and outdoor temperature conditions, humidity, wind speed, and solar radiation. Additionally, there is variation in the number of hidden layers and hidden neurons used in ANN models, indicating that there is no standard measure in designing ANN architecture to predict thermal conditions. Some models are simpler with only one hidden layer, while others are more complex with four hidden layers.

**Table 1:** Literature review of ANN-based models for predicting thermal conditions.

Papers	Input Features	Output Features	Number of Hidden Layers	Number of Hidden Neurons
Jin Woo Moon et al. [42]	- indoor temperature - changes in indoor temperature - outdoor temperature - cavity temperature - solar radiation - opening condition of inner surface - opening condition of outer surface	- indoor temperature	4	10
Jin Woo Moon et al. [40]	- outdoor temperature - changes in outdoor temperature - outdoor humidity - changes in outdoor humidity - indoor temperature - changes in indoor temperature - indoor humidity - changes in indoor humidity	- changes temperature - changes humidity	1	17
Arya Ashtiani et al. [43]	- sol-air temperature - wind speed - outdoor relative humidity - time of day	- indoor dry-bulb temperature	3	10
Markus Sulzer et al. [44]	- outdoor temperature - vapor pressure - mean sea level pressure - global irradiance - long-wave downwelling radiation - wind speed - solar altitude - solar azimuth - time of day - time of year - number of the day in week	- indoor temperature - indoor physiologically equivalent temperature	1	16
Weiwei Liu et al. [45]	- temperature - relative humidity - wind speed - mean radiation temperature	- thermal comfort level	1	5

Based on the discussion, a new model is proposed to predict and generate thermal condition parameter data in Rumoh Aceh, located in the Aceh region of Indonesia. This model is designed using the ANN algorithm. This model is tailored to the specific conditions of Rumoh Aceh, which may have unique characteristics different from the buildings studied in previous literature. Furthermore, this study also considers the influence of building orientation and position in predicting optimal thermal conditions, thus providing new insights into how geographic location and building orientation contribute to the thermal conditions in traditional buildings. The model training process utilizes input data and target data. The input data consists of meteorological data, including air temperature, humidity, duration of sunlight exposure, wind speed, and wind direction. Additionally, the target data comprises parameter data within the building, including temperature, humidity, and wind speed. These data were collected over the course of one month from five different rooms. The proposed model employs the ANN algorithm to predict thermal condition parameter data within rooms based on the building's position and orientation. This model is anticipated to aid in understanding and enhancing the thermal quality of Rumoh Aceh, by considering factors like building position and orientation as crucial elements in creating a comfortable and healthy living environment.

## 2 Materials and methods

### 2.1 Building design of Rumoh Aceh

Rumoh Aceh is a type of traditional Acehnese house with distinctive vernacular architecture, which means its design is adapted to the natural environment and climate of its surroundings. Rumoh Aceh is typically designed in an elevated platform form, a building raised from the ground surface with wooden columns as its supports. This raised platform design aims to protect the house from floods and maintain the building's stability in earthquake-prone areas [48]. The tall roof structure aids in maintaining good airflow within the house and reducing heat inside. Furthermore, sturdy wooden structures are employed as primary supports for the house. Natural ventilation is commonly integrated into Rumoh Aceh through windows that facilitate the flow of fresh air into the house. This ventilation system helps regulate the indoor temperature and creates a comfortable environment. Rumoh Aceh possesses a distinct and diverse room arrangement, depending on the house type and occupants' needs. Generally, the layout of rooms in Rumoh Aceh consists of various spaces such as the veranda, central living area, and bedrooms.

**Fig. 1:**
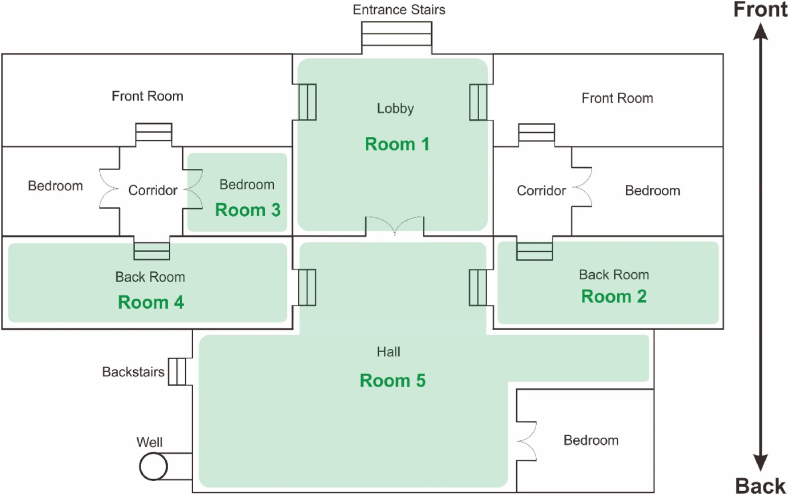
Location plan for measuring thermal condition of Rumoh Aceh.

According to ASTM C-1046 Standard, the optimal location for measurement instruments, such as the Heat Flux Transducer (HFT) and Temperature Transducer (TT), must be selected considering heat flow to ensure that the data obtained reflects the desired thermal conditions. Each location should exhibit one-dimensional heat flow to avoid errors from multi-dimensional heat flow that may affect the measurement results [49]. In this paper, the analyzed Rumoh Aceh consists of ten rooms that will be assessed and evaluated within the context of thermal conditions. There are five rooms selected for measurement purposes: lobby, bedrooms, backroom (left and right), and hall. Measurements at multiple points are conducted to obtain a comprehensive overview of data distribution related to the building's thermal conditions. For further clarity, please refer to Fig. 1. Subsequently, data from these five rooms are employed as sample datasets required for the ANN learning process. The thermal condition measurements include indoor temperature, humidity, and wind speed. Temperature and humidity sensors, specifically the DHT22 sensors, are placed within these five rooms. The DHT22 sensor has a temperature measurement accuracy of ±0.5 °C, while its humidity measurement accuracy is ±2–5% RH. These sensors are placed in every corner of the five rooms to ensure an even distribution of measurements. The sensors are positioned away from ventilation to avoid direct heat influence that could interfere with the measurement results. Each sensor is installed at a height of approximately 1.5 m from the floor to obtain measurements that are representative of thermal conditions at the human level. Wind speed sensors, using an anemometer, are placed in the hall to measure the airflow entering the building. This sensor has an accuracy of ±0.1 m/s. It is placed in the center of the building at a height of 1.5 m to capture the most representative wind speed as air enters the room. This placement is selected based on physical considerations that support data quality, including uniform heat distribution in each room, a representative distribution of thermal conditions, and accuracy in measuring airflow. With this approach, instruments are arranged to comprehensively capture variations in thermal conditions in each space, ensuring consistent data that aligns with ASTM C-1046 Standard.

All sensors were calibrated according to the manufacturer's standards before data collection. Measurements are taken every 30 min to collect sufficient data under various environmental conditions and to record daily temperature fluctuations due to weather changes or the sun's movement. This helps in modeling the thermal response of the house to changing environmental conditions throughout the day. This measurement data was then used as target data in the learning process. Additionally, in this study, radiant temperature was not measured. This is because the study focused solely on measuring thermal conditions inside the building, whereas radiant temperature is used to provide a more comprehensive understanding of thermal comfort as perceived by the human body.

The Qibla is the direction toward the Kaaba in Mecca, which serves as the direction for prayer for Muslims. This direction holds significant influence over the orientation of buildings in regions with a Muslim majority population. In the context of Islamic architecture, the orientation of a building considering the Qibla direction holds spiritual value. The Qibla position can impact the layout of rooms within a building. Rooms frequently used for prayer, such as personal worship spaces, are often arranged to face the Qibla. Additionally, an orientation that accounts for the Qibla position can also affect the use of other rooms. For instance, kitchens or bathrooms might be placed on the side opposite to the Qibla direction. Moreover, the province of Aceh is an area with a Muslim-majority population. The Qibla direction orientation based on the position of Rumoh Aceh discussed in this paper is 292.19°, which is 22.19° north of west. Building orientation has an impact on its thermal conditions. Proper orientation choices can optimize the use of natural energy sources such as sunlight and wind, contributing to the creation of a comfortable and efficient living environment. The influence of thermal conditions on building orientation is significant, as the building orientation affects how effectively a structure can utilize natural energy sources like sunlight and wind to regulate indoor temperature [[50], [51], [52]].

Appropriate building orientation can maximize the level of thermal conditions within the structure. A building facing the sun will absorb solar heat during daylight hours, naturally helping to warm up the interior. However, it's important to avoid excessive direct sunlight, especially in tropical regions, as it can cause the rooms to become overly hot [[53], [54], [55]]. The use of proper window coverings can aid in regulating the amount of sunlight entering the rooms. When a building is well-designed, outside air can enter and flow smoothly through windows or vents on the side of the building facing the dominant wind direction. This natural ventilation helps maintain good airflow and reduces humidity indoors, creating a cooler and more comfortable environment [56]. Building orientation can also provide protection against strong winds and rain. Structures facing the dominant wind direction can shield against strong gusts, reducing the risk of building damage [57,58]. Additionally, well-designed roofs and walls can provide protection against rain, creating a safer and more comfortable space.

### 2.2 Proposed ANN model

The ANN model will learn patterns from meteorological data and then utilize them to generate new data concerning the thermal conditions of Rumoh Aceh. The meteorological data used includes temperature, humidity, sunlight duration, wind speed, and direction. This data was obtained from the Meteorology, Climatology, and Geophysics Agency located in Banda Aceh, Aceh Province, Indonesia. Temperature and humidity measurements were conducted using a digital thermometer and hygrometer with accuracies of ±0.5 °C and ±2 % RH, respectively. Sunlight duration was measured using a calibrated pyranometer with an accuracy of ±5 %. Wind speed and direction were measured using an anemometer and weather vane with accuracies of ±0.1 m/s and ±5°, respectively. Data collection was conducted every 30 min throughout the day [59,60]. Through this learning process, the ANN model will be able to recognize complex interactions among various environmental factors and produce more accurate and relevant outcomes [61]. The structure of the proposed ANN model can be seen in Table 2.

**Table 2:** The proposed ANN-based models for predicting thermal conditions within the Rumoh Aceh building.

Purpose	Structure	Composition
Prediction of the thermal condition data including temperature and humidity within the Rumoh Aceh building. This network architecture is designed to generate MODEL_01		Input neurons: • maximum outdoor temperature •minimum outdoor temperature •average outdoor temperature •average outdoor humidity •duration of sunshine •maximum outdoor wind speed •direction outdoor wind speed •average outdoor wind speed Output neurons: •indoor temperature •indoor humidity Number of hidden neurons: •hidden layer 1: 32 neurons •hidden layer 2: 18 neurons Activation function: •hidden neurons: Sigmoid •output neuron: Rectified linear unit Training methods: •learning rate: 0.3 •momentum factor: 0.9 Number of datasets: 30
Prediction of the thermal condition data including wind speed within the Rumoh Aceh building. This network architecture is designed to generate MODEL_02		Input neurons: •maximum outdoor temperature •minimum outdoor temperature •average outdoor temperature •average outdoor humidity •duration of sunshine •maximum outdoor wind speed •direction outdoor wind speed •average outdoor wind speed Output neurons: •indoor wind speed Number of hidden neurons: •hidden layer 1: 32 neurons •hidden layer 2: 18 neurons Activation function: •hidden neurons: Sigmoid •output neuron: Rectified linear unit Training methods: •learning rate: 0.3 •momentum factor: 0.9 Number of datasets: 30

The thermal condition data used involves interactions among various environmental variables, making it nonlinear. This study also entails the collection and analysis of a large dataset, including thermal condition measurements from five different rooms over a one-month period. This data collection period was chosen as an initial step to understand the basic thermal condition patterns in Rumoh Aceh. Given that Indonesia is a tropical country, a one-month data collection period is considered optimal as it encompasses a variety of weather conditions. The ANN model was chosen due to its superior ability to learn and mimic complex patterns from large, nonlinear data, which aligns with the characteristics of the data used in this research. ANN learns from historical data to identify patterns and trends that may occur in the future. Additionally, ANN not only fits historical data but also learns patterns inherent in the data. This makes ANN an effective choice for predicting thermal conditions. To create the ANN model, a learning process is necessary. The ANN learning process involves two stages: Feed Forward and Back Propagation. In the Feed Forward stage, input data enters the network. Each neuron in the input layer receives input values and forwards them to the hidden layer. This signal progresses through the neurons until reaching the output layer. This process generates initial prediction data. Once initial prediction data is obtained, the Back Propagation stage begins. Here, a comparison between the predicted outcomes and the actual values of the target data is conducted. Error values are calculated and fed back into the network from the output layer to the input layer through existing connections. The goal of this stage is to adjust the weights and biases of each neuron, reducing the discrepancy between predictions and actual values [[62], [63], [64]]. The Feed Forward and Back Propagation processes occur iteratively until the generated errors become smaller, and the ANN model approaches the desired level of accuracy.

In this study, the dataset used consists of 30 data points collected from five different rooms for one month. Although the dataset size may seem small, there is strong justification for the ANN modeling. First, the data collected reflects significant daily variations in thermal parameters, providing a representation of different environmental conditions. Second, the ANN can learn from small datasets, helping to prevent overfitting. In previous studies, ANNs have been successfully used with small datasets to achieve accurate predictions in various environmental applications [61,65]. The dataset utilized corresponds to the month of September 2021. This dataset is divided into two main parts: input data and target data. The input data includes meteorological information related to the environmental conditions surrounding the building. This data was obtained from a weather station of the Meteorology, Climatology, and Geophysics Agency located at coordinates 5°31′20.69127″N 95°25′1.0185″E, which is approximately 16.04 km away from the Rumoh Aceh building located at coordinates 5°31′0.74393″N 95°16′19.08986″E. On the other hand, the target data includes direct measurement results pertaining to the thermal condition parameters within Rumoh Aceh. This target data is further divided into two subsets: target data for generating MODEL_01 and target data for generating MODEL_02. In MODEL_01, the target data used comprises the accumulation of daily average indoor temperature values and daily average indoor humidity values from the sampled data of five rooms. Conversely, in MODEL_02, the target data used involves the accumulation of daily average indoor wind speed values. Utilizing this dataset, the ANN model will learn the patterns of the relationship between meteorological data and thermal condition measurements. The model will be designed and trained to develop an understanding of how environmental factors influence the level of thermal quality within Rumoh Aceh. As such, the ANN model enables predictions of thermal conditions based on meteorological data at specific points in time.

The ANN model was designed using the Feedforward and BackPropagation methods, which are common and have proven effective in various predictive studies. Complete details about the computational process and network structure can be found in the literature [66], which has discussed this model.

### 2.3 Experimental method

Testing was conducted to analyze the accuracy of the proposed ANN model. The testing process involved calculating the error rate between the data generated by the ANN model and the original data obtained from measurements. Furthermore, testing was carried out to determine the optimal orientation of Rumoh Aceh buildings for thermal conditions. Additionally, the testing aimed to predict the level of thermal quality in Rumoh Aceh buildings in specific areas.

The optimal level of thermal quality is a state where individuals feel comfortable with the surrounding temperature, humidity, and wind speed conditions [67]. One common way to assess thermal conditions is by comparing temperature, humidity, and wind speed. Temperature measures the heat or coldness of the air around us. The range of temperatures considered comfortable varies depending on activities and environments, not being too low or too high. Meanwhile, humidity measures how much water vapor is present in the air compared to the maximum amount of water vapor the air can hold at a specific temperature without condensation occurring. Humidity is crucial in influencing human thermal comfort and various physical and chemical processes in the environment. High humidity tends to make the air feel uncomfortable due to dampness and sweating. Conversely, low humidity can cause the skin to feel dry. Wind speed also affects thermal conditions. Gentle wind can help maintain a comfortable body temperature, but strong winds can make the body feel colder and uncomfortable [[68], [69], [70], [71]].

**Fig. 2:**
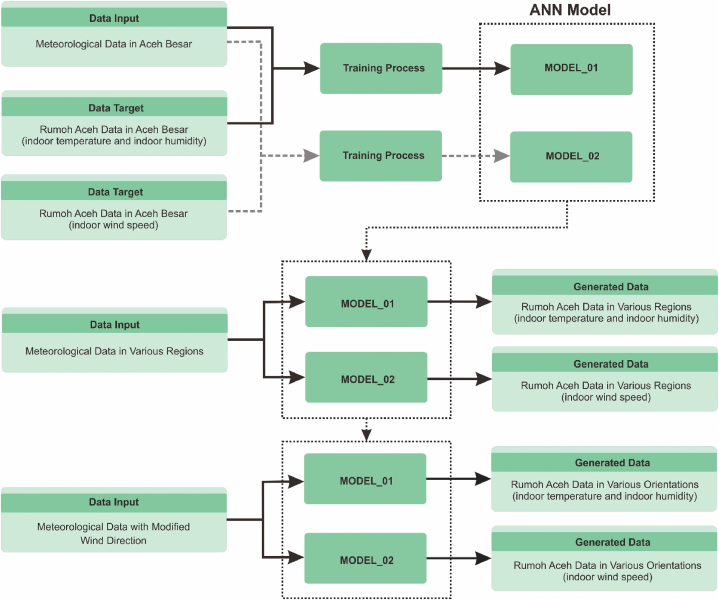
Experimental method for generated data from ANN model.

Fig. 2 depicts the testing diagram to generate data from the proposed ANN model. In this diagram, the ANN model is constructed through the learning phase. The resulting ANN models are MODEL_01 for predicting indoor data: temperature and humidity, and MODEL_02 for predicting indoor wind speed data. Once the ANN models are formed, they are used to generate new data that includes thermal condition parameters for different building orientations and positions. Output data related to building orientations are obtained through the modification of meteorological data related to varying wind speeds from different directions. Meanwhile, information about building positions is obtained from relevant meteorological data in the designated region.

In the first testing phase, data is generated by the previously constructed ANN model. Based on this data, an error analysis is conducted. The error analysis is performed to assess the accuracy level of the thermal condition prediction process for Rumoh Aceh. This analysis involves calculating the MAE (Mean Absolute Error) between the data generated by the ANN model and the original measured data. The data generated by the ANN model is the prediction output obtained under the same conditions and timeframe as the original measured data. The following equation is used to calculate the MAE,

(15) $$MAE=\frac{1}{n}\sum_{i=1}^{n}\left|x_{i}-{\hat{x}}_{i}\right|$$

where, n is the number of data points, $x_{i}$ represents the original measured data, and ${\hat{x}}_{i}$ represents the data generated by the ANN model.

**Fig. 3:**
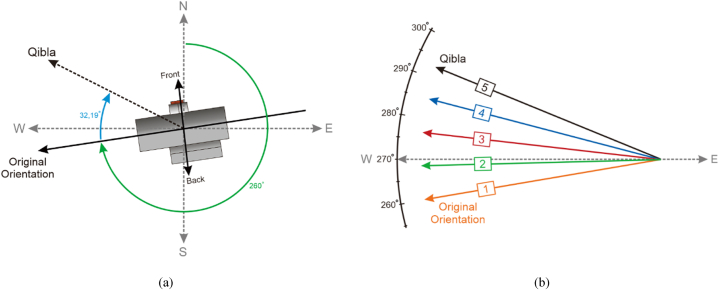
Experiment based on various orientations, (a) Orientation towards qibla direction, (b) Various orientation angle.

In the second testing phase, thermal condition data prediction for Rumoh Aceh is conducted based on variations in building orientations over a one-year period. As shown in Fig. 3(a), it represents the orientation of the Rumoh Aceh building discussed in this paper. The orientation of the Rumoh Aceh building is 260° to the west, with a difference of 32.19° from the qibla direction. Meanwhile, in Fig. 3(b), this testing includes five angular orientation positions with a difference of 8.05° between orientations. In Orientation 1, it is the original orientation of Rumoh Aceh with an angle value of 260°. Meanwhile, the angle values for Orientation 2 are 268.05°, for Orientation 3 are 276.1°, for Orientation 4 are 284.14°, and for Orientation 5 are 292.19°. The purpose of this testing is to analyze thermal condition data of the Rumoh Aceh building across different orientations. By comparing this thermal condition data, optimal building orientation that provides the highest level of thermal quality can be identified.

**Fig. 4:**
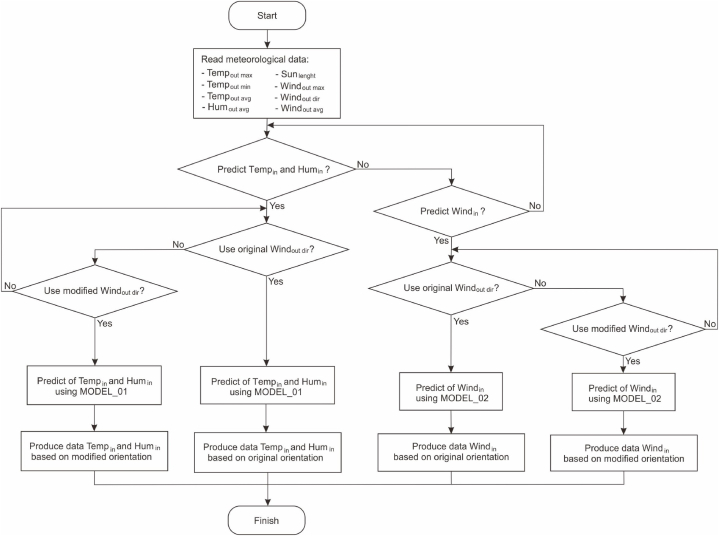
Flowchart to generate predictive data based on orientation.

The process of generating thermal condition prediction data based on building orientation is illustrated in the flowchart in Fig. 4. Prior to data generation, the first step involves preparing input data that includes meteorological data and the two ANN models: MODEL_01 and MODEL_02. MODEL_01 is used to predict indoor data: temperature and humidity, while MODEL_02 is used to predict indoor wind speed data. Subsequently, to generate data for various building orientations, modifications are made by altering the wind direction angle. This modification involves changing the value of the variable ${Wind}_{out\;dir}$ in the meteorological data. The next step involves utilizing each ANN model to predict and generate new data.

In the third testing phase, the thermal condition prediction data for Rumoh Aceh is generated based on variations in building positions in specific regions over a one-year period. This test involves four regions with different geographical positions, namely Sabang, North Aceh, and Nagan Raya. The initial position of the Rumoh Aceh building is in the Aceh Besar region. Then, for the test, the building's position is shifted to the Sabang region, 39.88 km to the north. For the next test, the building's position is shifted to the North Aceh region, 225.5 km to the east. In the final test, the building's position is shifted to the Nagan Raya region, 196.98 km to the southeast. The aim of this testing is to analyze the thermal condition data of the Rumoh Aceh building in various specific regions, thereby determining the most optimal level of thermal quality.

**Fig. 5:**
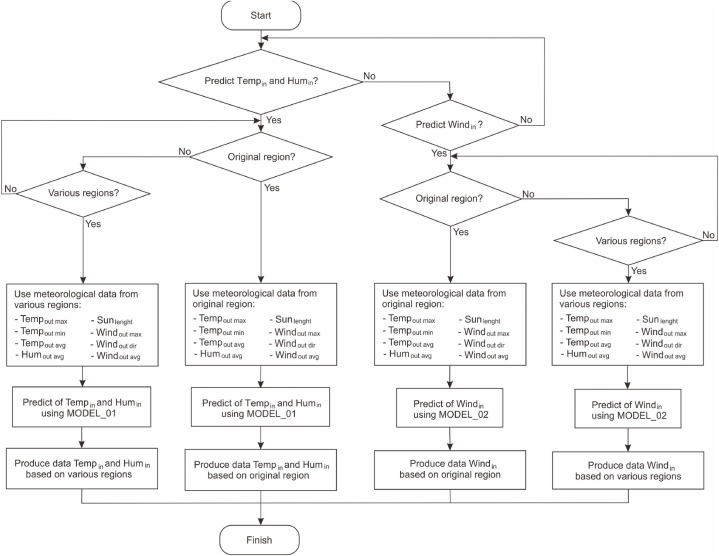
Flowchart to generate predictive data based on region.

The process of generating thermal condition prediction data based on building placement is illustrated in the flowchart in Fig. 5. Subsequently, to generate data based on variations in building locations, meteorological data from specific regions is required. For example, to predict the thermal comfort of Rumoh Aceh in the North Aceh region, meteorological data from North Aceh is needed, and a similar approach applies to other regions. The next step involves prediction using MODEL_01 and MODEL_02 according to their respective purposes.

## 3 Result and discussion

### 3.1 Analysis of ANN model performance

The process of ANN training results in two models: MODEL_01 and MODEL_02. These models are used to predict the thermal quality levels of Rumoh Aceh. This ANN model is employed to generate indoor data: temperature and humidity for each room (Room 1, Room 2, Room 3, Room 4, and Room 5), as well as indoor wind speed data.

In the subsequent process, testing is conducted to evaluate the performance of each obtained ANN model. Model performance evaluation involves calculating the discrepancies between the data generated by the ANN model and the original measurement data. The results of these discrepancies are presented in Table 3. Based on the test results conducted ten times, MODEL_01 obtained an overall average error value of 1.05. Specifically, the average error values in Room 1, Room 2, Room 3, Room 4, and Room 5 respectively are: 1.01, 1.16, 1.00, 1.06, and 1.02. Meanwhile, for MODEL_02, the average error value is 0.03. Thus, it can be concluded that the proposed ANN models possess an error tolerance of ±1.05 for MODEL_01 and an error tolerance of ±0.03 for MODEL_02.

**Table 3:** Error value between original data and generated data.

Experiment	Room 1		Room 2		Room 3		Room 4		Room 5		Wind Speed (m/s)
	T (°C)	Rh (%)	T (°C)	Rh (%)	T (°C)	Rh (%)	T (°C)	Rh (%)	T (°C)	Rh (%)	
1	0.15	0.73	0.65	0.48	0.40	0.05	0.04	0.46	1.76	0.13	0.11
2	0.04	0.56	1.25	0.03	0.02	0.00	0.19	0.01	1.79	0.07	0.01
3	0.11	1.25	0.19	3.56	0.26	5.26	0.37	5.89	0.76	0.02	0.01
4	1.08	0.13	0.54	0.06	1.36	0.01	1.23	0.03	0.08	0.00	0.01
5	0.07	0.16	0.02	1.02	0.63	1.06	0.62	0.13	0.46	0.68	0.01
6	0.06	5.15	0.11	3.18	0.04	1.44	0.16	1.42	0.54	5.87	0.02
7	0.34	0.31	0.48	0.00	0.99	0.10	0.39	0.21	0.06	0.19	0.10
8	0.67	1.63	0.81	1.27	1.32	1.22	0.66	1.19	0.26	0.03	0.00
9	0.02	1.27	0.24	2.18	0.01	0.03	0.29	0.55	0.16	0.45	0.01
10	1.24	5.24	1.58	5.46	2.43	3.34	1.59	5.80	1.27	5.88	0.06
**Max**	**1.24**	**5.24**	**1.58**	**5.46**	**2.43**	**5.26**	**1.59**	**5.89**	**1.79**	**5.88**	**0.11**
**Min**	**0.02**	**0.13**	**0.02**	**0.00**	**0.01**	**0.00**	**0.04**	**0.01**	**0.06**	**0.00**	**0.00**
**MAE**	**0.38**	**1.64**	**0.59**	**1.72**	**0.74**	**1.25**	**0.55**	**1.57**	**0.71**	**1.33**	**0.03**

T: Temperature; Rh: Relative humidity.

### 3.2 Analysis based on various orientations

**Fig. 6:**
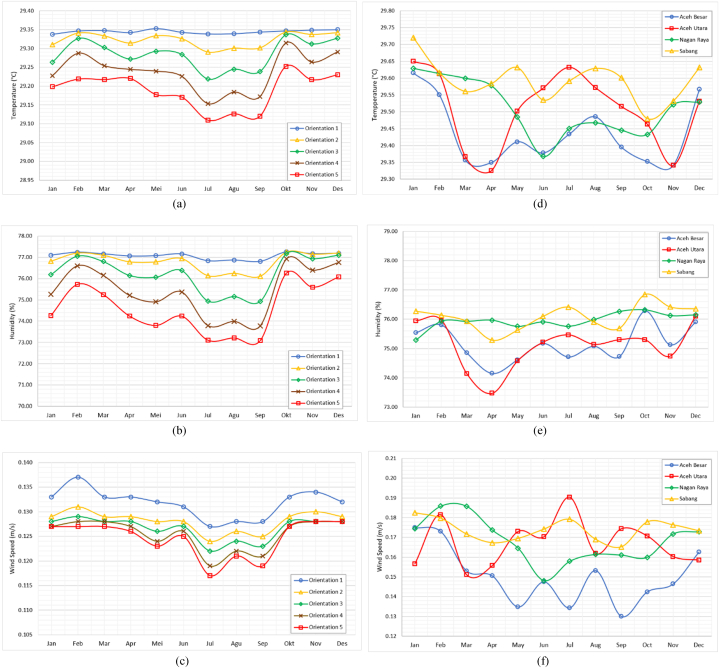
The comparison of thermal data in Rumoh Aceh; Based on various orientations: (a) indoor temperature, (b) indoor humidity, (c) indoor wind speed; Based on various regions: (d) indoor temperature, (e) indoor humidity, (f) indoor wind speed.

In Fig. 6(a), a comparison graph of indoor temperatures based on different orientations is presented. From the graph, it can be observed that Orientation 1 yields the highest indoor temperature data, with a range of approximately 29.34 °C–29.35 °C. This result tends to remain stable across each month. On the other hand, in Orientation 5, the lowest indoor temperature data is apparent, ranging around 29.11 °C–29.25 °C. The highest temperatures occur in February, October, November, and December, whereas the lowest temperatures are observed in August and September. Other findings also reveal that the highest temperature values are predominantly seen in October, while the lowest values are mostly prevalent in July. The data for July consistently produces the lowest temperatures across various building orientations due to the higher levels of rainfall during this month, reaching up to 34.3 mm.

Furthermore, in Fig. 6(b), a comparison graph of indoor humidity is shown. From the graph, it is evident that Orientation 1 yields the highest indoor humidity data, ranging approximately from 77.25 % to 76.81 %. Conversely, in Orientation 5, the lowest indoor humidity data is generated, with a range of around 73.09 %–76.25 %. This finding reveals that the highest humidity values are predominantly observed in October, while the lowest humidity values are mostly prevalent in September.

Next, in Fig. 6(c), a comparison graph of indoor wind speed is displayed. As before, it is evident that Orientation 1 produces the highest indoor wind speed data, ranging between 0.127 m/s and 0.137 m/s, while Orientation 5 yields the lowest indoor wind speed data, ranging between 0.117 m/s and 0.128 m/s. These findings also reveal that the highest wind speed values tend to occur in February, November, and December, while the lowest wind speed values tend to occur in July.

In Table 4, the accumulation of average values for indoor: temperature, humidity, and wind speed in each building orientation is presented. The results of this analysis conclude that Rumoh Aceh in Orientation 5 provides the most optimal thermal quality from the perspective of temperature and humidity. The lowest average values are found in Orientation 5, with a temperature of 29.19 °C and humidity of 74.57 %. However, in terms of wind speed, Orientation 5 has the lowest value at 0.125 m/s. Meanwhile, the highest value is observed in Orientation 1, which is 0.132 m/s. Additionally, in another finding, the building's orientation in Orientation 5 aligns with the qibla direction. This finding also has cultural and local wisdom implications in Aceh. Buildings that face the qibla direction hold significant religious meaning, especially within the context of building and architecture. Therefore, these results not only indicate optimal thermal conditions but also reflect alignment with the religious and cultural values of the local community.

**Table 4:** The mean thermal value of Rumoh Aceh based on building orientations and regions.

Parameters	Features	Indoor Temperature (°C)	Indoor Humidity (%)	Indoor Wind Speed (m/s)
Orientations	1	29.34	77.07	0.132
	2	29.32	76.81	0.128
	3	29.29	76.23	0.127
	4	29.24	75.42	0.125
	5	29.19	74.57	0.125
Regions	Aceh Besar	29.44	75.16	0.150
	North Aceh	29.51	75.11	0.167
	Nagan Raya	29.51	75.95	0.168
	Sabang	29.59	76.08	0.174

### 3.3 Analysis based on various regions

In Fig. 6(d), a comparison graph of indoor temperatures based on the placement regions of the buildings is shown. In the Aceh Besar region, indoor temperatures range from 29.34 °C to 29.62 °C, in the North Aceh region they range from 29.33 °C to 29.65 °C, in the Nagan Raya region they range from 29.37 °C to 29.63 °C, and in the Sabang region they range from 29.48 °C to 29.72 °C. Based on the graph, it can be observed that the highest temperatures across different regions tend to occur in January. Meanwhile, the lowest temperatures vary in each region. In Aceh Besar, it occurs in November; in North Aceh, it occurs in April; in Nagan Raya, it occurs in June; and in Sabang, it occurs in October.

Furthermore, in Fig. 6(e), a comparison graph of indoor humidity based on the placement regions of the buildings is presented. In the Aceh Besar region, indoor humidity ranges from 74.15 % to 76.28 %, in the North Aceh region it ranges from 73.47 % to 76.11 %, in the Nagan Raya region it ranges from 75.29 % to 76.32 %, and in the Sabang region it ranges from 76.28 % to 76.85 %. From the graph, it can be observed that the highest humidity levels across different regions tend to vary each month. In Aceh Besar and North Aceh regions, it occurs in April; while in Nagan Raya and Sabang regions, it occurs in January. Additionally, the lowest humidity levels also vary across each region. In Aceh Besar, Nagan Raya, and Sabang regions, it occurs in October; while in North Aceh region, it occurs in December.

Next, in Fig. 6(f), a comparison graph of indoor wind speed based on the placement regions of the buildings is displayed. In the Aceh Besar region, indoor wind speed ranges from 0.130 m/s to 0.175 m/s, in the North Aceh region it ranges from 0.151 m/s to 0.190 m/s, in the Nagan Raya region it ranges from 0.148 m/s to 0.186 m/s, and in the Sabang region it ranges from 0.165 m/s to 0.183 m/s. From the graph, it is evident that the highest wind speed levels across different regions tend to vary each month. In Aceh Besar and Sabang regions, it occurs in January; in the North Aceh region, it occurs in July; and in the Nagan Raya region, it occurs in February and March. Additionally, the lowest wind speed levels also vary across each region. In Aceh Besar and Sabang regions, it occurs in September; in the North Aceh region, it occurs in March; and in the Nagan Raya region, it occurs in June.

In Table 4, the cumulative average values of indoor: temperature, humidity, and wind speed for each building placement region are presented. The results of this analysis reveal that the Aceh Besar region has the lowest average temperature value, at 29.44 °C. North Aceh has the lowest average humidity value, at 75.11 %, and Sabang has the highest average wind speed value, at 0.174 m/s. These findings indicate that the three regions: Aceh Besar, North Aceh, and Sabang, possess optimal thermal quality levels for Rumoh Aceh buildings.

## 4 Conclusion

Based on the results of the conducted analysis, it can be concluded that using the ANN algorithm as a method to develop ANN models proves to be effective in understanding the complex interactions between environmental variables and thermal condition variables within buildings. In this study, two models were generated: MODEL_01, for predicting indoor temperature and humidity, and MODEL_02, for predicting indoor wind speed. Both of these models demonstrated the ability to generate data with a tolerance error level of ±1.05 for MODEL_01 and ± 0.03 for MODEL_02. Furthermore, the analysis results also indicate that building orientation at an angle of 292.19°, facing the Qibla, produces the most optimal thermal quality in terms of temperature and humidity. This result also holds cultural and local wisdom significance in Aceh. Meanwhile, building orientation at an angle of 260° achieves thermal quality supported by a better wind speed factor. The analysis results also reveal variations in indoor: temperature, humidity, and wind speed values depending on specific locations and times (January to December). Therefore, when designing buildings, geographical location factors need to be considered to achieve optimal thermal quality.

This study successfully developed a predictive model using ANN to forecast thermal conditions in Rumoh Aceh buildings, guiding planners and architects in designing structures that create comfortable living spaces while considering traditional aspects and local wisdom. The resulting model demonstrated good and consistent performance for all tested building orientations, as there were no significant differences in thermal condition parameters between orientations. However, this study has limitations, as the tested building orientations do not encompass all possible variations in the field, meaning that these results need further testing under more diverse conditions to ensure the model's generalization. Additionally, this study did not consider other variables such as building materials, structure, and room layout. Further research is needed to address these limitations by testing the model across a wider range of orientations and considering additional aspects.

## CRediT authorship contribution statement

**Muslimsyah:** Project administration, Conceptualization, Project administration, Conceptualization. **Abdul Munir:** Validation, Investigation. **Yuwaldi Away:** Writing – review & editing, Validation, Conceptualization. **Abdullah:** Validation, Investigation, Validation, Investigation. **Teuku Yuliar Arif:** Funding acquisition, Formal analysis. **Andri Novandri:** Writing – original draft, Resources, Methodology, Formal analysis, Data curation, Conceptualization.

## Data availability statement

The authors do not have permission to share data.

## Declaration of competing interest

The authors declare that they have no known competing financial interests or personal relationships that could have appeared to influence the work reported in this paper.
